# Clinical Differences and Outcomes of COVID-19 Associated Pulmonary Thromboembolism in Comparison with Non-COVID-19 Pulmonary Thromboembolism

**DOI:** 10.3390/jcm11206011

**Published:** 2022-10-12

**Authors:** Santiago de Cossio, Diana Paredes-Ruiz, Covadonga Gómez-Cuervo, Jesús González-Olmedo, Antonio Lalueza, Yolanda Revilla, Carlos Lumbreras, Carmen Díaz-Pedroche

**Affiliations:** 1 Department of Internal Medicine, 12 de Octubre University Hospital, 28041 Madrid, Spain; 2 Radiology Department, 12 de Octubre University Hospital, 28041 Madrid, Spain

**Keywords:** COVID-19, pulmonary thromboembolism, risk factors, signs and symptoms, recurrence, bleeding

## Abstract

(1) Background: Severe acute respiratory syndrome coronavirus 2 (SARS-CoV-2) infection has been reported to increase the risk of pulmonary thromboembolism (PTE). The aim of this study is to elucidate whether Coronavirus disease COVID-19-associated PTE has a different clinical expression than non-COVID-19 PTE due to a different pathophysiology. (2) Methods: retrospective study of PTE episodes conducted at our hospital between January 2019 and December 2020, comparing the group of COVID-19-associated PTE patients with a control group of non-COVID-19 PTE patients. (3) Results: A total of 229 patients with PTE were registered, 79 of whom had COVID-19. Cancer (15.2% vs. 39.3%; *p* < 0.001), previous surgery (0% vs. 8%; *p* = 0.01), previous VTE (2.5% vs. 15.3%; *p* = 0.003), signs and/or symptoms of deep venous thrombosis (DVT) (7.6% vs. 22.7%; *p* = 0.004) and syncope (1.3% vs. 8.1%; *p* = 0.035) were less frequent in the COVID-19 group. Central thrombosis was more frequent in the control group (35.3% vs. 13.9%; *p* = 0.001). No VTE recurrent episodes were observed in the COVID-19 group, whereas four (2.7%) episodes were recorded for the control group. One-month bleeding rate was higher in the COVID-19 group (10.1% vs. 1.3%; *p* = 0.004). (4) Conclusion: COVID-19-associated PTE has clinical characteristics that differ from those of PTE without COVID-19, including inferior severity and a lower rate of VTE recurrence. Physicians should be aware of the high risk of bleeding in the first month of COVID-19-associated PTE.

## 1. Introduction

Severe acute respiratory syndrome coronavirus 2 (SARS-CoV-2) infection has been reported to increase thrombotic risk. In fact, a prothrombotic state, represented by serum D-dimer elevation, is independently correlated with mortality in COVID-19 patients [1,2,3,4,5], and different anticoagulation regimens have been successfully tested [3,6,7,8]. A higher incidence of pulmonary thromboembolism (PTE) in the context of COVID-19, compared to control cohorts [9,10,11], might suggest that other thrombotic mechanisms underlie beyond the classic venous thromboembolism (VTE) risk factors [12]. In 1884, Virchow proposed that thrombosis is the result of at least one of three underlying etiologic factors: vascular endothelial damage, stasis of the blood flow and blood hypercoagulability [13]. COVID-19 necropsies have reported endothelial inflammation and microthrombosis in the pulmonary circulation despite the use of prophylactic anticoagulation, described as immunothrombosis [14,15,16,17,18]. Thus, two of the three Virchow’s etiologic factors involving the pulmonary circulation can be observed in the context of COVID-19: endothelial damage and hypercoagulability. Thrombosis in the pulmonary circulation appears because of these phenomena. Moreover, thrombosis causes an impairment of the oxygen exchange process [12,19], which might increase the severity of COVID-19. The term pulmonary thrombosis (PT), in the context of COVID-19-associated PTE, has appeared recently in the literature [12,20], highlighting the increasing relevance of in situ thrombosis in this disease, which differs from non-COVID-19-PTE, commonly caused by the migration of a thrombus generally located in the lower limbs. Whether this different pathophysiology has a different clinical expression is not completely clear [16,21,22]. Severely ill COVID-19 patients and those admitted to intensive care units (ICU) have shown a higher incidence of PTE, but the prognosis was not affected by the presence of PTE in this group of severely affected patients [11,23,24,25]. Indeed, imaging-based findings describing less frequent parameters of right ventricle overload, more peripheric distribution and a lower thrombus load in COVID-19-associated PTE might indicate an inferior PTE-related severity [22,24,26,27]. In this study, we compare the COVID-19-associated PTE (COVID-19 group) with the non-COVID-19 PTE (control group) patients, focusing on clinical presentation characteristics and prognosis. Our hypothesis is that COVID-19-associated PTE’s different pathophysiology might carry along differences in terms of the clinical characteristics, PTE-related severity and mid-term prognosis.

## 2. Materials and Methods

All PTE events in adult patients admitted to the “12 de Octubre” University Hospital (Madrid, Spain) between January 2019 and December 2020 were retrospectively registered. Two groups were stablished depending on whether the PTE event took place in the context of SARS-CoV-2 infection (COVID-19 group) or not (control group). PTE diagnosis was stablished by computed tomography pulmonary angiography (CTPA) or ventilation–perfusion scintigraphy (VPS). SARS-CoV-2 infection was confirmed by polymerase chain reaction (PCR). From the beginning of the COVID-19 pandemic, patients included in the control group were required to have a negative SARS-CoV-2 test upon admission and to have not been infected with COVID-19 or been vaccinated in the previous three months.

Patients were followed up for twelve months. We recorded anthropometric variables, previous conditions, VTE risk factors, including active cancer (defined as newly diagnosed cancer or cancer undergoing treatment); clinical signs and symptoms at the moment of PTE suspicion, including the heart rate, systolic blood pressure and signs and symptoms of VTE; electrocardiographic changes and CTPA parameters, including the most proximal thrombus location; the presence of right ventricle overload parameters in echocardiogram or in CTPA; the treatment duration, mortality and bleeding rate at one, three and twelve months; and VTE recurrence after one year. Bleeding was defined as major following the International Society on Thrombosis and Haemostasias definition [28]. Recurrent DVT was defined as a new noncompressible vein segment or an increase in the vein diameter by at least 4 mm compared with the last available measurement acquired by venous ultrasonography. Recurrent PTE was defined as a new ventilation–perfusion disparity or new intraluminal filling defect detected upon relevant imaging [29].

Quantitative variables were expressed using means with the standard deviation (SD) or median with the interquartile range (IQR) and compared using Student’s *t*-test for independent samples or the Mann-Whitney U test, as appropriate. The Kolmogorov-Smirnov test was employed to test the normality of the distribution. Qualitative variables were expressed as absolute and relative frequencies and compared by χ^2^ or Fisher’s exact tests. A *p*-value of <0.05 was considered significant. 

Statistical analyses were performed using SPSS version 23 (IBM, Armonk, NY, USA).

All patients or their health care providers provided written or oral consent for their participation in the registry in accordance with the local ethics committee requirements. The study was approved by the local ethics committee of the hospital.

The aim of the present study was to assess whether COVID-19-associated PTE presents with different clinical characteristics and outcomes than non-COVID PTE due to its different pathophysiology.

## 3. Results

A total of 229 patients diagnosed with PTE were included in the study. Overall, there were 79 in the COVID-19 group and 150 in the control group. Their baseline characteristics are shown in Table 1. Both groups were similar in terms of age, sex, body mass index and cardiovascular risk factors.

In the group of COVID-19-associated PTE patients, at the moment of diagnosis, 34 (60.6%) patients were breathing room air, 11 (19.7%) were receiving a fraction of inspired oxygen (FiO_2_) ranging between 0.24 and 0.4 mmHg, and 11 (19.7%) were receiving FiO_2_ at levels higher than 0.4 mmHg. ICU admission was required by 16 (20.3%) patients.

Regarding the classic risk factors of VTE, cancer disease (15.2% vs. 39.3%; *p* < 0.001), surgery in the previous two months (0.0% vs. 8.0%; *p* = 0.01) and a previous history of VTE (2.5% vs. 15.3%; *p* = 0.003) were less frequent in the COVID-19 group. The only factor that was more frequent in the COVID-19 group was a bedridden status longer than three days.

Signs or symptoms of DVT (7.6% vs. 22.7%; *p* = 0.004) were less frequent in the COVID-19 group. In the control group, syncope was more frequently reported than in the COVID-19 group (8.1% vs. 1.3%; *p* = 0.035), in addition to negative T-waves inEKG (19.0% vs. 3.4%; *p* = 0.004). However, although there was a tendency in the control group to present more frequently hypotension, a statistically significant difference was not reached (12.8% vs. 6.3%; *p* = 0.13). No differences were reported regarding the presence of new-onset chest pain, cough, dyspnea and hemoptysis.

In terms of the imaging technique results, central or main PTE (35.3% vs. 13.9%; *p* = 0.001) and lobar PTE (30.1% vs. 14.6%; *p* = 0.001) were more frequent in the control group than in the COVID-19 group, whereas segmental (48.1% vs. 30.1%; *p* = 0.008) and subsegmental PTE (23.4% vs. 7.7%; *p* = 0.001) were more frequent in the COVID-19 group. Right ventricle overload parameters in CT or echocardiography were similar between groups (24.1% vs. 28.0%, *p* > 0.2). Abnormal chest radiography results were more frequent in the COVID-19 group (88.0% vs. 65.0%; *p* = 0.001). At one-year follow-up, 55 patients in the COVID-19 group and 126 in the control group were alive, comprising 81.0% and 82.0%, respectively (*p* > 0.2). One-month mortality was higher in the COVID-19 group (12.7% vs. 4.0%; *p* = 0.015), and mortality between the second and third month was higher in the control group (9.7% vs. 2.6%; *p* = 0.05). No VTE recurrent episodes were observed in the COVID-19 group after one year, whereas 4 (2.7%) episodes were recorded in the control group, without statistically significant differences (*p* = 0.143). Anticoagulant treatment duration was similar between groups (140 vs. 153 median days; *p* > 0.2). Bleeding in the first month was statistically more frequent in the COVID-19 group (10.1% vs. 1.3%; *p* = 0.004). No differences were observed in the second-to-third-month bleeding rate (0% vs. 2.0%; *p* > 0.2) and twelve-month bleeding rate (10.1% vs. 6.0%; *p* > 0.2). All bleeding events in the COVID-19 group took place in the first month, and half of them (50%) were major bleeding events. Major bleeding rate at one year was similar in both groups (5.1% vs. 3.3%; *p* > 0.2).

## 4. Discussion

The primary goal of our study was to identify differences between COVID-19-associated PTE and PTE without COVID-19. To answer this question, we performed a retrospective study examining the episodes of PTE registered at our hospital in 2019 and 2020. The main classifying variable was the presence of SARS-CoV-2 infection as a possible determinant of the PTE episode. The strength of our study is based on the fact that it includes not only radiological parameters but also clinical characteristics and a one-year follow-up, with results in terms of mortality, bleeding rate and VTE recurrence. This is, to the best our knowledge, the first report that includes these parameters of follow-up and the largest cohort, with information regarding signs and symptoms of VTE.

The outcomes of our COVD-19-associated PTE group, with a one-month mortality rate of 10.2%, ICU admission of 20.3% and a need for supplementary oxygen therapy of 39.4% at the moment of PTE suspicion, are similar to those reported in previous studies, although a wide variety of findings have been described [4,11,26].

Our results showed that COVID-19-associated PTE has characteristics that might differ from those of PTE without COVID-19. Classic VTE risk factors, signs and symptoms of DVT, the main pulmonary artery thrombosis or central thrombus location and electrocardiographic alterations were significantly less frequent in the COVID-19 group. These results support the hypothesis of previous reports, stating that PTE in the context of COVID-19 might be a different phenotype of VTE [22]. However, in contrast to our results, these previous reports described similar clinical characteristics in both groups.

The presence of classic VTE risk factors and signs and symptoms of DVT are generally related to the embolism of a thrombus originating in the peripheric venous system. In our study, only bedridden status was more frequent in the COVID-19 group. This result is probably overestimated due to the mandatory isolation requirements during the first waves of the pandemic. Apart from that, the lesser frequency of these parameters in the COVID-19 group supports the hypothesis of a different pathophysiology, in which local prothrombotic conditions play a role beyond the effects of the classic VTE risk factors [14,15,16,17,18]. In fact, van Dam et al. [22] stated that all the pulmonary segments affected by thrombosis also showed COVID-19 radiological specific changes, which highlights the importance of local prothrombotic conditions in COVID-19-related PTE. The absence of differences in common symptoms related to the presence of pulmonary involvement, such as dyspnea, new-onset chest pain and hemoptysis, which were observed in our study, support this hypothesis. On the other hand, obesity in COVID-19 patients has been reported to increase the possibility of developing a PTE, and statin therapy might be a protective factor [27]. These results might indicate that, although their relevance is likely inferior, the classic mechanisms and risk factors of VTE still have an impact on COVID-19-associated PTE. This question might seem to be a theoretical matter, but some authors argue in favor of the possibility of a beneficial effect of antiplatelet therapy in COVID-19-associated PTE [30] based on results that showed an increased platelet count in arterial thrombosis compared with pulmonary emboli [31]. Their hypothesis asserts that pulmonary microthrombosis behaves similarly to arterial thrombosis; therefore, platelets must play a major role in the pathogenesis of pulmonary microthrombosis [30]. However, in the RECOVERY trial [32], antiplatelet therapy did not reduce mortality rates in patients hospitalized with COVID-19. The authors described a reduction in thrombotic events, although there were no differences in terms of the PTE incidence [32].

With respect to the thrombus location, our results agree with previous reports that identified a more peripherical distribution of the thrombus in the context of COVID-19 compared to a control PTE group [22,26,27,33]. Similar to the findings previously reported [22,27], the rate of centrally located thrombosis in our study was around 15%. A higher rate of central PTE (63.4%) associated with COVID-19 was reported in only one study (*n* = 47). Thus, further research is needed [34].

Regarding the PTE severity, previous studies conducted outside the context of COVID-19 suggest that syncope and main pulmonary artery involvement might be related to a higher hemodynamic instability and right ventricle disfunction [19,35,36]. Although we observed no differences in the right ventricle overload parameters based on imaging techniques; in our study, syncope, right ventricle overload electrocardiographic changes and main pulmonary artery thrombosis were significantly less frequent in the COVID-19 group. These latter results agree with those of previous authors, suggesting that COVID-19-associated PTE involves less PTE-related severity than PTE without COVID-19 [22,24]. In fact, there is some evidence suggesting that PTE’s coexistence with DVT might be related to an increased severity of PTE [37]. Therefore, the inferior rate of limb pain or edema registered in the COVID-19 group could also be an indirect indicator of a lower severity.

One-month mortality was statistically higher in the COVID-19-associated PTE group. In fact, a mortality sub-analysis identified SARS-CoV-2 infection as a statistically significant independent predictor of one-month mortality. In contrast, second-to-third-month mortality was lower in the COVID-19 group, and no differences were found in one-year mortality. These results probably reflect the reduced PTE-related severity of COVID-19-associated PTE compared to the non-COVID-19 PTE and the loss of virulence of COVID-19 after the end of the acute phase of the disease.

Remarkably, no episodes of VTE recurrence were registered among the 79 patients of the COVID-19 group in comparison to a 2.7% recurrence rate in the control group. As no episodes of recurrent VTE associated with COVID-19 occurred, no information regarding the management of VTE recurrence was provided.

One-month bleeding rate was also statistically higherin the COVID-19 group. In fact, all bleeding events in this group occurred in the first month. No differences in the one-year bleeding rate were found. Fernandez-Capitan et al. [4] reported a major bleeding rate of 3.2% in COVID-19-associated PTE at ten-day follow up, which is comparable to the 5.1% rate at the one-month follow-up recorded in our study. There are no previous reports that compare the bleeding rates between COVID-19-associated PTE and non-COVID-19 PTE. However, the higher one-month bleeding rate in the COVID-19 group obtained in our study might indicate that COVID-19-related coagulopathy could not only increase the thrombotic risk but also increase the bleeding risk.

Currently, anticoagulation for at least three months is the mainstay treatment for VTE associated with COVID-19 in the absence of other major risk factors, following the recommendations of the general VTE guidelines [19]. Thus, if this inferior recurrence rate were to be confirmed, it would up open the possibility of exploring shorter anticoagulant regimens for COVID-19-associated PTE, which might reduce the bleeding events. Considering the fact that around 70% of the COVID-19-associated PTE cases observed in our study were segmental (48.1%) or subsegmental (23.4%), and that no episodes of recurrence occurred, we recommend a close monitoring of anticoagulation during the first month of COVID-19-associated PTE and its termination after three months if no other reasons for continuing are presented, 

Although this is one of the largest cohorts describing the clinical presentation characteristics of PTE related to COVID-19, our study is not without limitations. It is based on a relatively limited number of patients, and it is a single-institution retrospective study.

## 5. Conclusions

There is evidence supporting the relevance of local thrombotic factors in the context of COVID-19 that are different from those of the standard VTE, leading to a different phenotype of the disease. However, it is more likely that COVID-19-associated PTE is the result of the combination of both in situ immunothrombosis and classic VTE risk factors.

Due to this different clinical phenotype, the diagnostic ability of the PTE clinical probability scales might be reduced, as they consider the presence of signs and symptoms of DVT to establish the risk of these events [19]. Further research should be performed in order to answer this question.

More studies are needed to confirm our hypothesis of a minor severity of COVID-19-associated PTE and a less significant rate of VTE recurrence.

COVID-19 associated coagulopathy might be related with the increased bleeding rate observed during the first month after PTE. Thus, in this context, we advocate the obtainment of a firm diagnosis in order to initiate therapeutic anticoagulant doses, followed by close monitoring of anticoagulation and the termination of anticoagulation at three months if no other reasons for continuing are presented.

## Figures and Tables

**Table 1:** Characteristics of pulmonary thromboembolism associated with COVID-19 vs. pulmonary thromboembolism without COVID-19.

Variable	COVID-19 Group (*n* = 79)	Control Group (*n* = 150)	*p*-Value
*Baseline characteristics*			
Age (years)—median (IQR)	61 (32)	67 (31)	
Male sex—n (%)	44 (55.7%)	84 (56.0%)	
Body mass index—median (IQR)	25.8 (7.1)	27.4 (6.9)	
Hypertension—n (%)	31 (39.2%)	59 (39.9%)	
Type-2 diabetes—n (%)	10 (12.7%)	21 (14.1%)	
Chronic heart failure—n (%)	5 (6.3%)	8 (5.4%)	
Atrial fibrillation—n (%)	6 (7.6%)	4 (2.7%)	
COPD—n (%)	5 (6.3%)	13 (8.7%)	
Cancer disease—n (%)	12 (15.2%)	59 (39.3%)	*p* < 0.001
Previous surgery (2 months)—n (%)	0 (0%)	12 (8.0%)	*p* = 0.01
Bedridden > 3 days—n (%)	72 (92.3%)	41 (27.5%)	*p* < 0.001
Previous VTE—n (%)	2 (2.5%)	23 (15.3%)	*p* = 0.003
Family history VTE—n (%)	2 (2.8%)	6 (4.1%)	
*Clinical findings*			
Signs or symptoms of DVT—n (%)	6 (7.6%)	34 (22.7%)	*p* = 0.004
Unilateral lower limb pain—n (%)	4 (5.1%)	26 (17.4%)	*p* = 0.008
Unilateral lower limb oedema—n (%)	5 (6.3%)	32 (21.3%)	*p* = 0.003
Hemoptysis—n (%)	4 (5.1%)	7 (4.7%)	
Dyspnea—n (%)	60 (75.9%)	103 (69.1%)	
Syncope—n (%)	1 (1.3%)	12 (8.1%)	*p* = 0.035
Chest pain (new onset)—n (%)	32 (40.5%)	72 (48.3%)	
Fever—n (%)	26 (32.9%)	26 (17.3%)	*p* = 0.007
Cough—n (%)	27 (34.6%)	42 (28.2%)	
Heart rate (bpm)—median (IQR)	94 (25)	94 (30)	
Systolic blood pressure (mmHg)—median (IQR)	123 (27)	128 (28)	
Hypotension (SAP < 100 mmHg)—n (%)	5 (6.3%)	19 (12.8%)	
Respiratory rate (breaths per minute)—median (IQR)	24 (12)	24 (11)	
*Complementary tests*			
EKG findings of right ventricle overload	12 (15.2%)	37 (24.7%)	*p* = 0.096
Right branch block—n (%)	5 (8.3%)	6 (4.9%)	
S1Q3T3—n (%)	6 (10.2%)	12 (9.8%)	
Negative T wave in precordial leads	2 (3.4%)	23 (19.0%)	*p* = 0.004
Abnormal chest-X-ray—n (%)	59 (88.1%)	80 (65.0%)	*p* = 0.001
Right ventricle overload (TTE or CT)	19 (24.1%)	42 (28.0%)	
Main pulmonary artery or central location—n (%)	11 (13.9%)	53 (35.3%)	*p* = 0.001
Lobar PTE—n (%)	13 (14.6%)	43 (30.1%)	*p* = 0.001
Segmental PTE—n (%)	37 (48.1%)	43 (30.1%)	*p* = 0.008
Subsegmental PTE—n (%)	18 (23.4%)	11 (7.7%)	*p* = 0.001
*Anticoagulant treatment*			
Treatment duration (days)—median (IQR)	140 (172)	154 (145)	
*Mortality*			
1-year mortality—n (%)	15 (19.0%)	27 (18.0%)	
1-month mortality—n (%)	10 (12.7%)	6 (4.0%)	*p* = 0.015
2nd to 3rd month mortality—n (%)	2 (2.5%)	14 (9.7%)	*p* = 0.05
*Recurrence*			
1-year recurrence—n (%)	0 (0.0%)	4 (2.7%)	
*Bleeding*			
1-year bleeding—n (%)	8 (10.1%)	9 (6.0%)	
Major bleeding—n (%)	4 (5.1%)	5 (3.3%)	
1-month bleeding—n (%)	8 (10.1%)	2 (1.3%)	*p* = 0.004
2-to-3-month bleeding—n (%)	0 (0.0%)	3 (2.0%)	

Data are presented as No. (%), median (interquartile range (IQR)) or mean  ±  standard deviation (SD). COPD: chronic obstructive pulmonary disease; CT: computed tomography; DVT: deep venous thrombosis; EKG: electrocardiogram; PTE: pulmonary thromboembolism; SAP: systolic arterial pressure; TTE: transthoracic echocardiography; VTE: venous thromboembolism.

## Data Availability

S.d.C. and C.D.-P. have full access to the data and are the guarantors of the data.
